# Departments Which Do Not Always Pay

**Published:** 1912-05-04

**Authors:** 


### INSTITUTIONAL HOUSEKEEPING.

### Departments Which Do Not Always Pay.

### II.—BREAD AND CONFECTIONERY.

In a vast number of establishments bread is the staff of life. Whether the bread be made on the premises or contracted for from a baker, the bakery is an important department demanding unceasing supervision. It is the bread and butter at breakfast, tea, and supper, on which healthy appetites are satisfied, and it happens not seldom that they are thankful to fall back upon bread and cheese to end their dinner likewise. This being so, it becomes a matter of the utmost importance to provide that the bread in institutions is appetising and nourishing. Some experiments recently made by an eminent physiologist are worthy of the attention of all institution managers. He first fed a number of pigeons for a certain period on white bread, with the result that they were reduced to the point of death by starvation. Other pigeons meantime fed on "standard" and on wholemeal bread remained well nourished and in good condition. A very weak and slightly acid solution was then made from grains of wheat and a teaspoonful of this water was mixed with the dying pigeons' food. An immediate improvement took place, and by adding this extract of the outer covering of the wheat, so weak that it defied chemical analysis, to the white bread, it became sufficiently nourishing to sustain life. Such experiments would seem to show that there is a grave loss in white bread as usually prepared, and that whatever the price paid for it, the hospital bakery department which is supplied with white bread only does not pay.

The case for the institutional bakery rests on the difficulty of controlling the quality of the flour used in contracts for bread. When complaints arise that members of the staff are ill-nourished, it is far more often that the flour is at fault than the meat, although it is the latter which attracts most attention from house committees. Needless to say, the hospital bakery needs expert supervision quite as much as the laundry. It is not to be supposed that the matron will have sufficient leisure to give the daily attention necessary to insure that good supplies of flour are bought and properly stored; that scrupulous cleanliness is observed; and that the right quantities of bread are ready at the right moment. But an efficient housekeeper, trained in the art of bread-making, would find this an interesting department in which substantial economies could be made.

The principal expenses of the bakery are wages, fuel, and outlay on plant. The latter is not a serious one, though the bakery for a large institution demands a properly constructed oven and good accommodation both for the mixing and storing of the bread. Nor is fuel a serious item, since it is mainly coke and hard coal which is required. Wages are undoubtedly a considerable expense. Where the numbers run into hundreds and the bakery assumes large proportions, it is necessary to engage a man, as the manual labour is too heavy for females; but for medium-sized establishments it will be found sufficient to engage a strong kitchen-maid to undertake this branch of work under the direction of the cook and housekeeper, one or both of whom should have practical knowledge of the subject. It will then be found that the saving effected in the price of the loaf will meet all the other outgoings.

Home-made bread need not be made every day, and there remains spare labour which may be utilised in other directions if there be a clever housekeeper with ideas in her head. Gluten and diabetic breads and biscuits are made in the Guy's Hospital bakery. The monotony of institution menus is wonderfully relieved by well-made and wholesome cakes, pastry, and other delicacies, which are too apt if they appear at all on the table to prove a stumbling-block to digestion in the hands of the hospital plain cook. All sorts of crisp, sweet, good things can be produced at very small cost in the home bakery under the direction of a bakeress who is encouraged to take pains.

The housekeeper who had at her disposal an extra maid could plenish her store-cupboards at very small cost by undertaking home-made preserves. Marmalades and jams made in bulk and stored in big tins are astonishingly cheaper and of better value than the bought articles. Pickles, too, and preserved fruit and vegetables can be very easily made at home, as every old-fashioned cook used to know. The surplus from the garden or from subscribers' gardens can be turned to very good account in such ways, and if labour is all that stands in the way it may be remembered that extra help of an unskilled nature is pitifully cheap, and that for a few shillings a week one or two respectable girls could, under direction, get through work worth many pounds to the hospital.

When the housekeeper's department is raised to one of dignity and responsibility, subject to and in no way infringing on the matron's prerogative, many admirable developments will become possible. It is only under such conditions that the departments which we have outlined can be made to pay.

---

### HOUSEKEEPING QUERIES.

Puzzled.—Young cooks invariably complain of their ovens, and we are not surprised that yours has so much difficulty, even though you say it is an excellent modern range. Ascertain if the dampers are in working order and are thoroughly understood, and at the next complaint that the oven will not heat or brown foods investigate the condition of the flues and of the spaces over and under the ovens: in all likelihood, through laziness or ignorance, they are choked with soot. To heat the oven, the top of the fire must be kept clear and powerful, in order to draw the flames over and round the iron box. Put on lumps of coal mixed with a little coke or a few cinders level with the top of the oven, but not with the iron plates that form the top of the stove. Pull out the dampers from the oven, in order to create a draught, and make up the fire frequently, adding only a little at a time. But the biggest fire will not heat an oven which is coated round with such a non-conductor of heat as soot.

